# Supplementary material for: Fine-scale modelling finds that breeding site fragmentation can reduce mosquito population persistence
Source: Commun Biol. 2019 Jul 25;2:273. doi: 10.1038/s42003-019-0525-0 (PMC6658551; doi:10.1038/s42003-019-0525-0)
Supplement: Supplementary file 1 — Supplementary Information [file 42003_2019_525_MOESM1_ESM.pdf]

Supplementary Material

Supplementary Figures

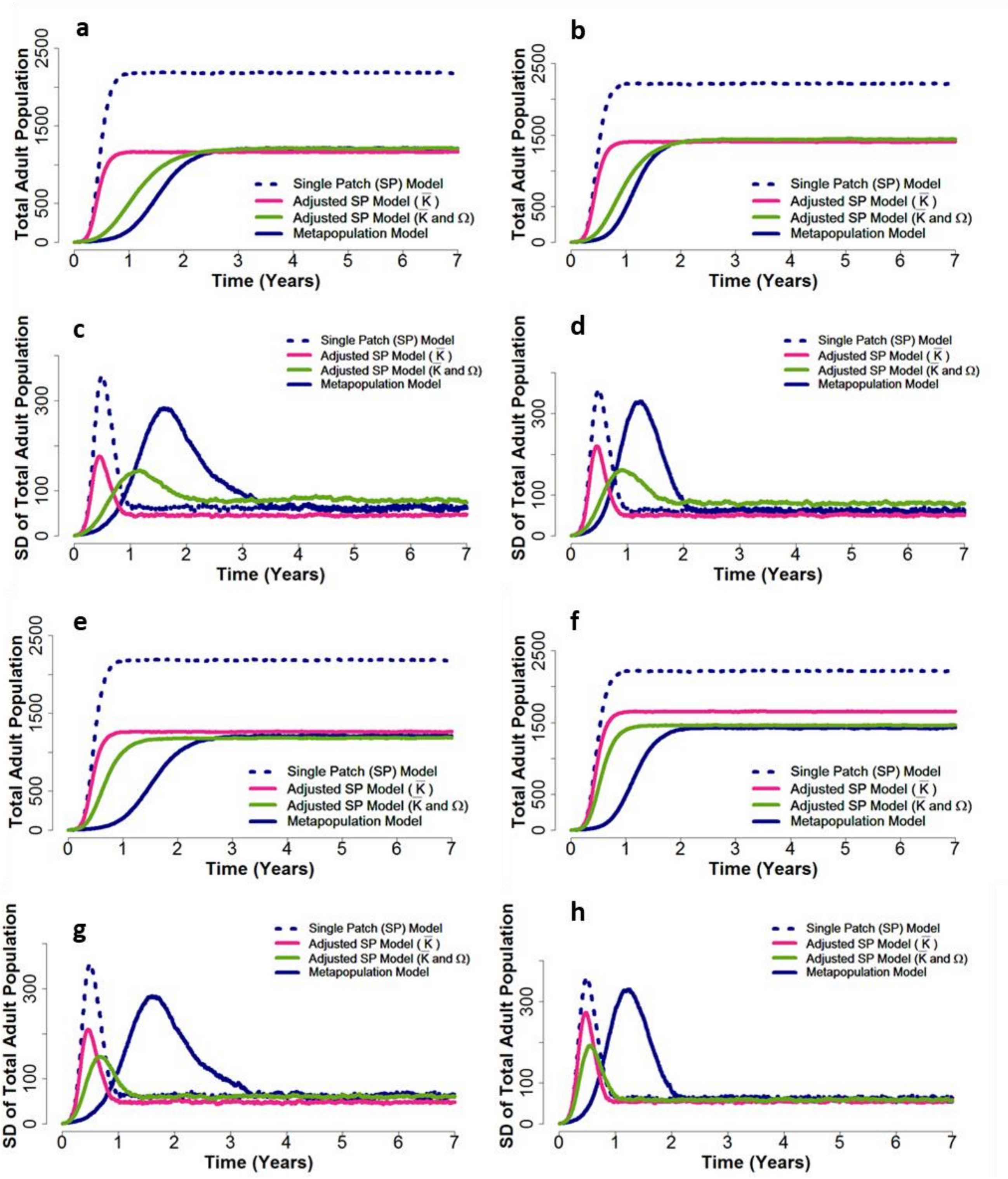

Supplementary Figure 1: Single Patch Approximations - Example 1 Comparison of results obtained when we approximate the invasion dynamics observed when a homogeneous landscape comprised of 1024 patches, with an equilibrium larval population of 3 larvae per patch, is seeded with 3 adult mosquitoes. A mean dispersal length of 5 patches and for a dispersal rate of 0.10 (a,c,e,g) and 0.30 (b,d,f,h) was used. (a-d): This corresponds to the scenario where we approximate two quantities - the mean equilibrium total adult mosquito population and the growth rate of the population. (e-h): Here we approximate three quantities - the mean and variance of the equilibrium total adult mosquito population, and the growth rate of the population. For each scenario, the mean and variance were calculated across 1000 realisations of the stochastic model.

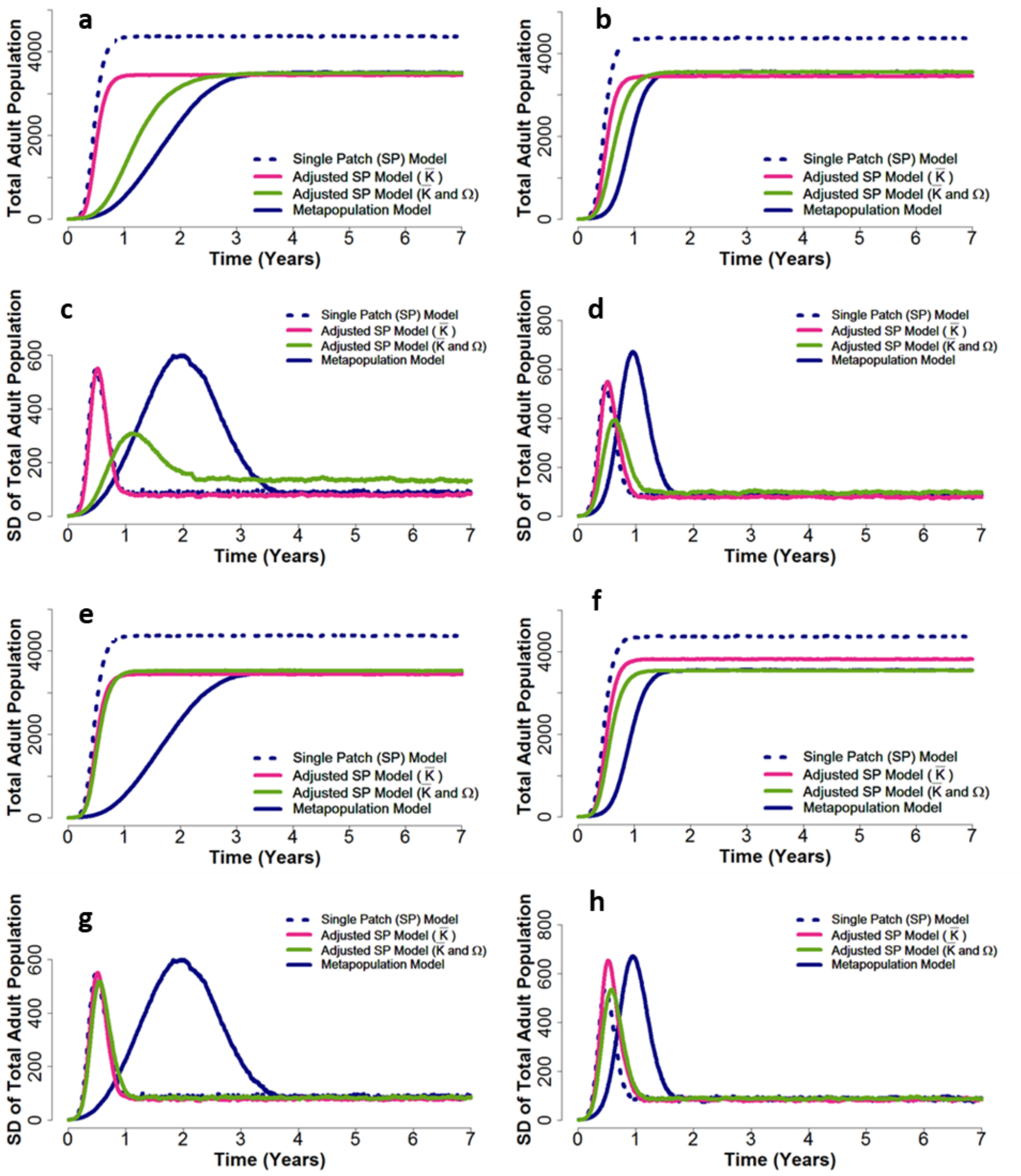

Supplementary Figure 2: Single Patch Approximations - Example 2 Comparison of results obtained when we approximate the invasion dynamics observed when a homogeneous landscape comprised of 1024 patches, with an equilibrium larval population of 6 larvae per patch, is seeded with 6 adult mosquitoes. A dispersal rate of 0.1 and a mean dispersal length of 1 patch (a,c,e,g) and 5 patches (b,d,f,h) was used. (a-d): This corresponds to the scenario where we approximate two quantities - the mean equilibrium total adult mosquito population and the growth rate of the population. (e-h): Here we approximate three quantities - the mean and variance of the equilibrium total adult mosquito population, and the growth rate of the population. For each scenario, the mean and variance were calculated across 1000 realisations of the stochastic model.

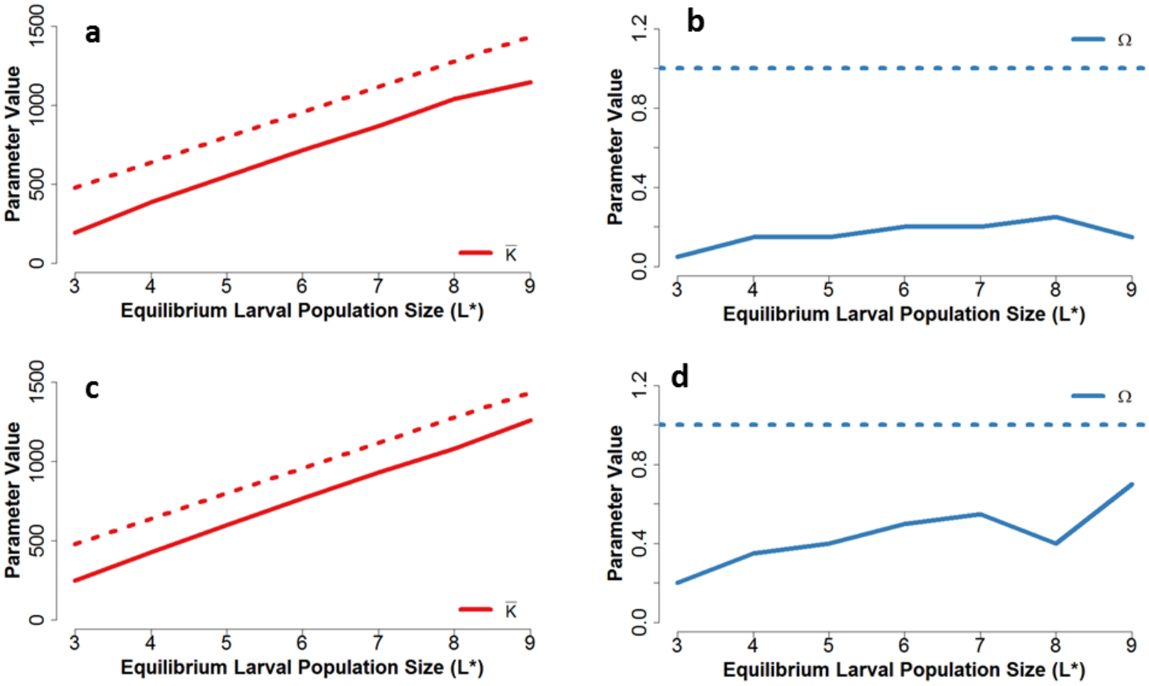

Supplementary Figure 3: Single Patch Approximations - Magnitude of Adjustment. Example of difference between the best fitting values of $\overline{K}$ and Ω in the adjusted single patch model (solid lines) and the corresponding values in the unadjusted single patch model. For each scenario, 1000 realisations of the stochastic model were performed.

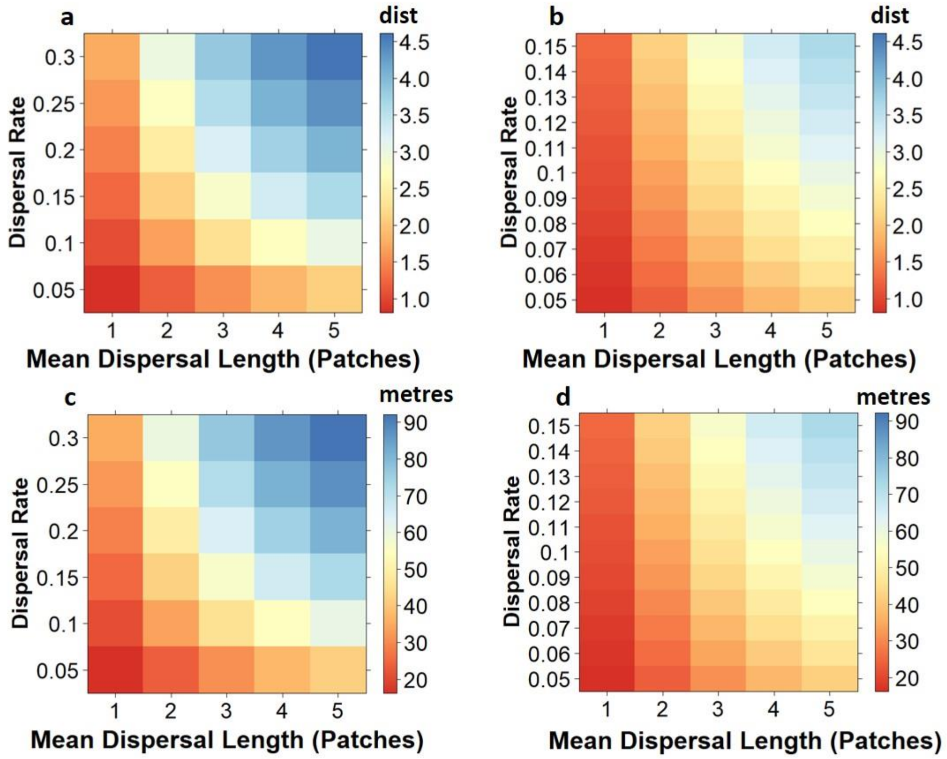

Supplementary Figure 4: Mean Lifetime Dispersal Distance Comparison of the estimated mean lifetime dispersal distance of adult mosquitoes for different values of the daily dispersal rate and mean dispersal length per day, for a homogeneous landscape on a 32x32 grid. This was calculated as described in equation (18). Figures a and b show estimates in terms of Euclidean distance between patches, and Figures c and d show estimates in terms of metres (assuming each patch in our 32x32 grid represents an area of approximately 20mx20m). For each scenario, the mean and variance were calculated across 1000 realisations of the stochastic model.
